# Supplementary material for: IDO1+ Paneth cells promote immune escape of colorectal cancer
Source: Commun Biol. 2020 May 22;3:252. doi: 10.1038/s42003-020-0989-y (PMC7244549; doi:10.1038/s42003-020-0989-y)
Supplement: Supplementary file 1 — Supplementary Information [file 42003_2020_989_MOESM1_ESM.pdf]

Supplementary figures

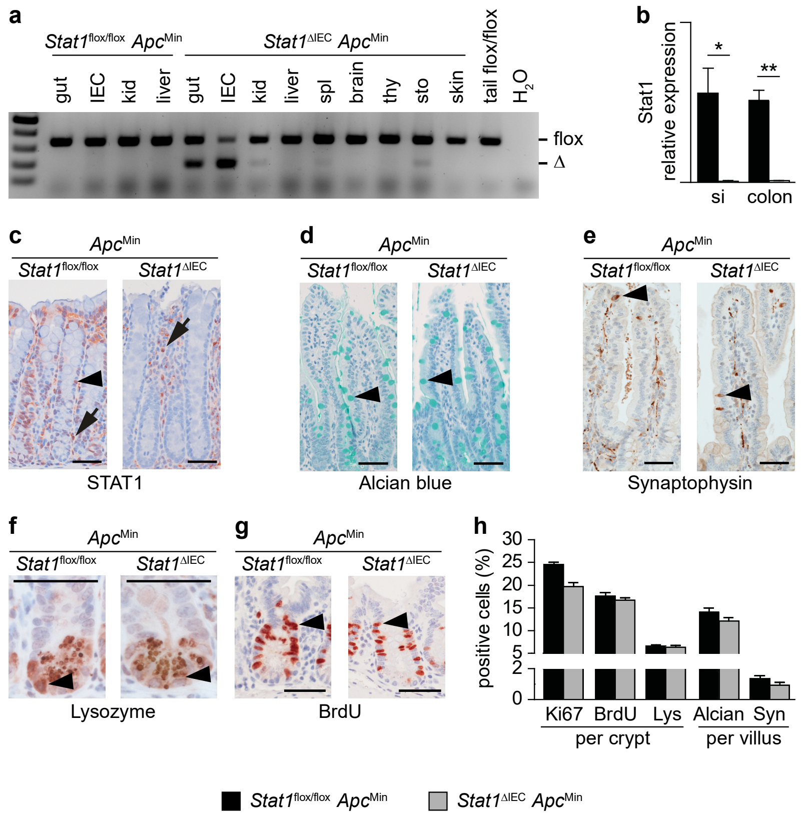

Supplementary figure 1: Intestinal cell differentiation is not affected in *Stat1*$^{\Delta IEC}$ *Apc*^{Min} mice. (a) PCR for conditional deletion of *Stat1* in different organs and cells of *Stat1*$^{\Delta IEC}$ *Apc*^{Min} mice. IEC: intestinal epithelial cells; kid: kidney; spl: spleen; thy; thymus; sto: stomach. (b) qPCR for Stat1 mRNA expression in isolated epithelial cell preparations from si and colon (*Stat1*$^{flox/flox}$ *Apc*^{Min} si n = 4, colon n = 5; *Stat1*$^{\Delta IEC}$ *Apc*^{Min} si n = 4, colon n = 5). (c) IHC staining of the colon for total STAT1. STAT1^{+} epithelial cells are indicated by arrowheads in *Stat1*$^{flox/flox}$ *Apc*^{Min} mice. Lamina propria cells stained positive for STAT1 in both genotypes (arrows). Scale bars indicate 50µm. (d-h) Characterization of mucosal cell types. Goblet cells were stained with Alcian blue (d), enteroendocrine cells with IHC for Synaptophysin (e), Paneth cells with IHC for Lysozyme (f) and proliferating cells with IHC for Ki67 (not shown) and BrdU incorporation (g) in the small intestine of *Stat1*$^{flox/flox}$ *Apc*^{Min} and *Stat1*$^{\Delta IEC}$ *Apc*^{Min} mice. Corresponding cell types are indicated by arrowheads. Scale bars indicate 50µm. (h) Quantitation of mucosal cell types using stained sections (*Stat1*$^{flox/flox}$ *Apc*^{Min} Ki67 16 crypts, 2 mice; BrdU 21 crypts, 3 mice; Lys 19 crypts, 2 mice; Alcian 15 villi, 2 mice; Syn 20 villi, 2 mice; *Stat1*$^{\Delta IEC}$ *Apc*^{Min} Ki67 15 crypts, 2 mice; BrdU 23 crypts, 3 mice; Lys 15 crypts, 2 mice; Alcian 15 villi, 2 mice; Syn 23 villi, 2 mice). si: small intestine. Bars represent mean +/- SEM.

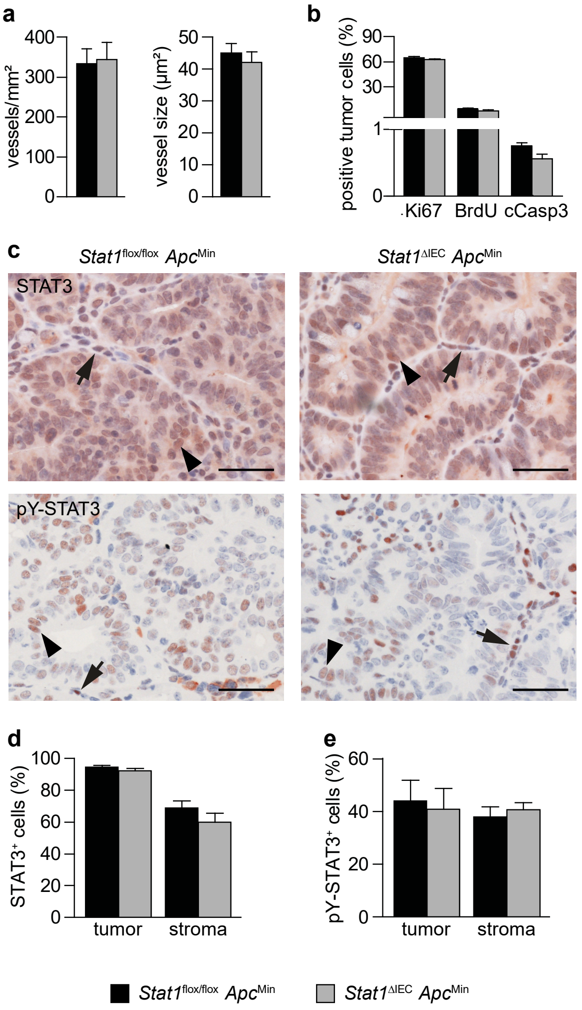

Supplementary figure 2: Vessel formation and Stat3 activation are not affected in tumors of *Stat1*$^{\Delta IEC}$ *Apc*^{Min} mice. (a) Assessment of blood vessel parameters (vessel density and vessel size) in the stroma of intestinal *Stat1*$^{flox/flox}$ *Apc*^{Min} and *Stat1*$^{\Delta IEC}$ *Apc*^{Min} tumors with IHC staining for Endomucin (*Stat1*$^{flox/flox}$ *Apc*^{Min} 28 tumors, 5 mice; *Stat1*$^{\Delta IEC}$ *Apc*^{Min} 32 tumors, 5 mice). (b) Quantitation of cell proliferation (Ki67 and BrdU) and apoptosis (cleaved Caspase 3) in tumors of *Stat1*$^{flox/flox}$ *Apc*^{Min} and *Stat1*$^{\Delta IEC}$ *Apc*^{Min} mice using IHC-stained sections (*Stat1*$^{flox/flox}$ *Apc*^{Min} Ki67 25 tumors, 6 mice; BrdU 29 tumors, 5 mice; cCasp3 25 tumors, 6 mice; *Stat1*$^{\Delta IEC}$ *Apc*^{Min} Ki67 26 tumors, 5 mice; BrdU 25 tumors, 8 mice; cCasp3 24 tumors, 5 mice). (c) IHC staining for STAT3 and Tyr705-phosphorylated STAT3 (pY-STAT3) in intestinal tumors of *Stat1*$^{flox/flox}$ *Apc*^{Min} and *Stat1*$^{\Delta IEC}$ *Apc*^{Min} mice. Tumor cells (arrowheads) and stroma cells (arrows) showed expression and activation of STAT3. Scale bars indicate 50µm. (d, e) Histomorphometric quantitation of STAT3^{+} (d) and pY-STAT3^{+} (e) tumor and stroma cells in *Stat1*$^{flox/flox}$ *Apc*^{Min} and *Stat1*$^{\Delta IEC}$ *Apc*^{Min} tumors (STAT3: *Stat1*$^{flox/flox}$ *Apc*^{Min} tumor 24 tumors, 5 mice; *Stat1*$^{\Delta IEC}$ *Apc*^{Min} tumor 25 tumors, 5 mice; *Stat1*$^{flox/flox}$ *Apc*^{Min} stroma 23 tumors, 5 mice; *Stat1*$^{\Delta IEC}$ *Apc*^{Min} stroma 25 tumors, 6 mice; pY-STAT3: tumor 24 tumors, 5 mice each; *Stat1*$^{flox/flox}$ *Apc*^{Min} stroma 24 tumors, 6 mice; *Stat1*$^{\Delta IEC}$ *Apc*^{Min} stroma 24 tumors, 5 mice). Bars represent mean +/- SEM.

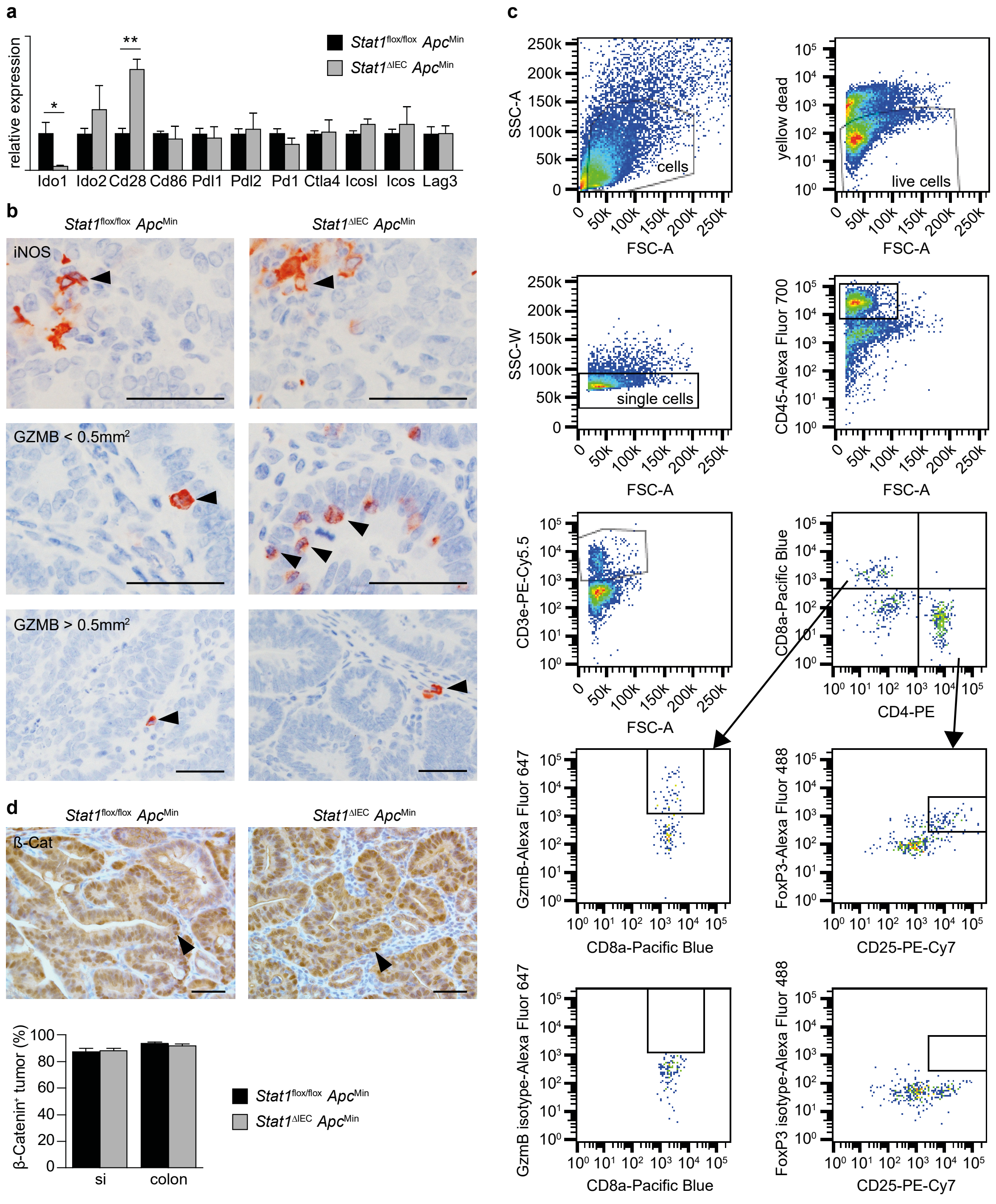

Supplementary figure 3: Reduced Ido1 and increased CD28 expression in tumors of *Stat1*$^{\Delta IEC}$ *Apc*^{Min} mice. (a) Expression of immune checkpoints and immune stimulatory molecules in *Stat1*$^{flox/flox}$ *Apc*^{Min} and *Stat1*$^{\Delta IEC}$ *Apc*^{Min} tumors assessed by RNA-seq. (b) IHC staining of immune cell infiltrates (arrowheads) in tumors. Scale bars indicate 50µm. (c) Gating strategies for FACS analysis of immune cells isolated from *Stat1*$^{flox/flox}$ *Apc*^{Min} and *Stat1*$^{\Delta IEC}$ *Apc*^{Min} tumors. FSC: forward scatter; SSC: side scatter. (d) IHC staining for ß-Catenin in *Stat1*$^{flox/flox}$ *Apc*^{Min} and *Stat1*$^{\Delta IEC}$ *Apc*^{Min} tumors. Positive nuclei of tumor cells are indicated by arrowheads. Scale bars indicate 50µm. Histomorphometric quantitation of nuclear β-Catenin in *Stat1*$^{flox/flox}$ *Apc*^{Min} (si 12 tumors, 4 mice; colon 12 tumors, 5 mice) and *Stat1*$^{\Delta IEC}$ *Apc*^{Min} (si 12 tumors, 4 mice; colon 12 tumors, 5 mice) tumors. si: small intestine. Bars represent mean +/- SEM.

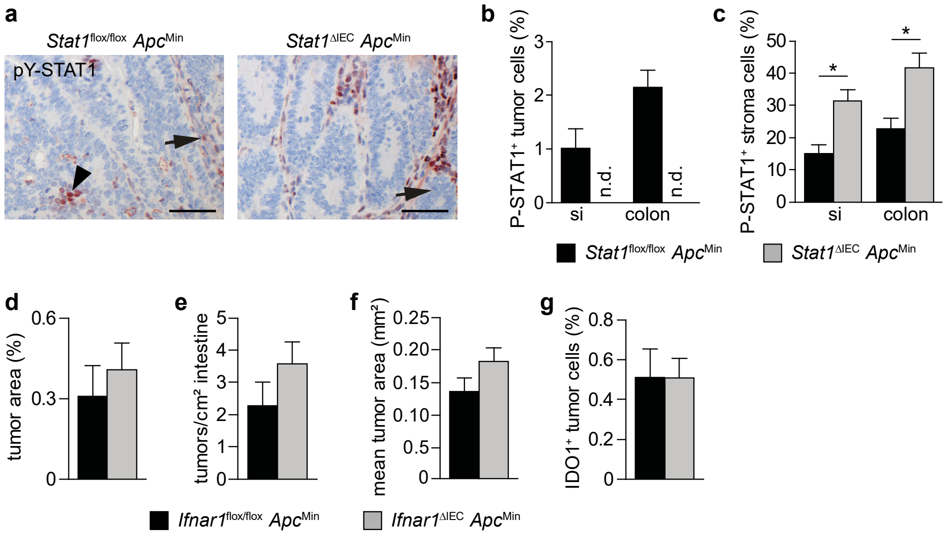

Supplementary figure 4: Type I IFN signaling is not required for formation of IDO1$^+$ Paneth cells in *Apc*^{Min} tumors. (a) IHC staining of *Stat1*$^{flox/flox}$ *Apc*^{Min} and *Stat1*$^{\Delta IEC}$ *Apc*^{Min} tumors for Tyr701-phosphorylated pY-STAT1. Positive tumor cells in *Stat1*$^{flox/flox}$ *Apc*^{Min} tumors are indicated by arrowheads. Stroma cells are indicated by arrows. Scale bars indicate 50µm. (b, c) Quantitation of pY-STAT1$^+$ tumor (b) and stroma cells (c) in *Stat1*$^{flox/flox}$ *Apc*^{Min} and *Stat1*$^{\Delta IEC}$ *Apc*^{Min} tumors (*Stat1*$^{flox/flox}$ *Apc*^{Min} tumor si 16 tumors, 7 mice; colon 20 tumors, 5 mice; *Stat1*$^{\Delta IEC}$ *Apc*^{Min} tumor si 12 tumors, 4 mice; colon, 12 tumors, 4 mice; *Stat1*$^{flox/flox}$ *Apc*^{Min} stroma si 12 tumors, 4 mice; colon 12 tumors, 5 mice; *Stat1*$^{\Delta IEC}$ *Apc*^{Min} stroma si 12 tumors, 4 mice; colon 12 tumors, 4 mice). (d-f) Tumor formation in *Ifnar1*$^{flox/flox}$ *Apc*^{Min} (n = 8) and *Ifnar1*$^{\Delta IEC}$ *Apc*^{Min} (n = 18) mice. (g) Quantitation of IDO1$^+$ tumor cells in *Ifnar1*$^{flox/flox}$ *Apc*^{Min} (10 tumors, 8 mice) and *Ifnar1*$^{\Delta IEC}$ *Apc*^{Min} (14 tumors, 10 mice) tumors using IHC-stained sections. si: small intestine; n.d.: not detectable. Bars represent mean +/- SEM.

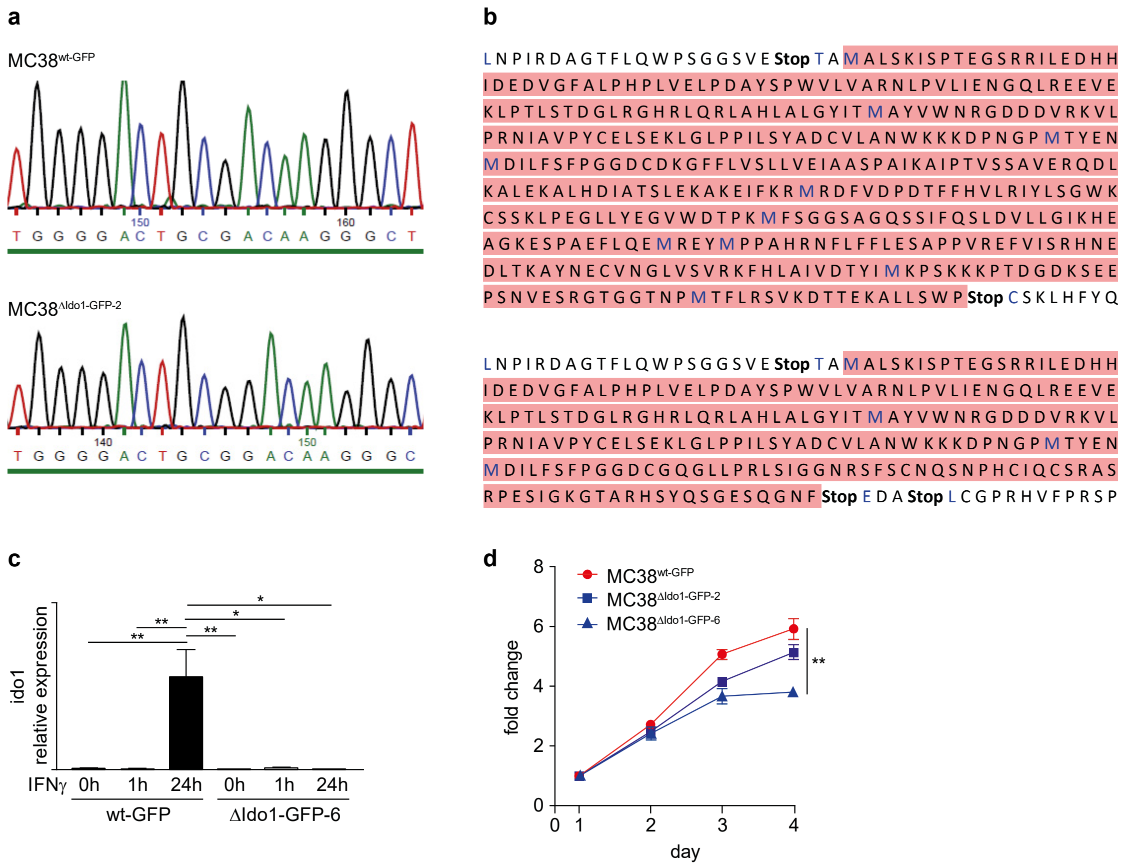

Supplementary figure 5: Characterization of MC38$^{\Delta Ido1\text{-}GFP}$ cells. (a, b) Sequence analyses (a) and predicted coding sequences (b) of MC38$^{wt\text{-}GFP}$ and MC38$^{\Delta Ido1\text{-}GFP\text{-}2}$ *Ido1* target sites. (c) qPCR for Ido1 mRNA expression in MC38$^{wt\text{-}GFP}$ and MC38$^{\Delta Ido1\text{-}GFP\text{-}6}$ cells 0, 1 and 24 hours after IFNγ stimulation (n = 3). (d) Growth curves of MC38$^{wt\text{-}GFP}$ and MC38$^{\Delta Ido1\text{-}GFP}$ cells (3 replicates, representative of two experiments). Bars represent mean +/- SEM.

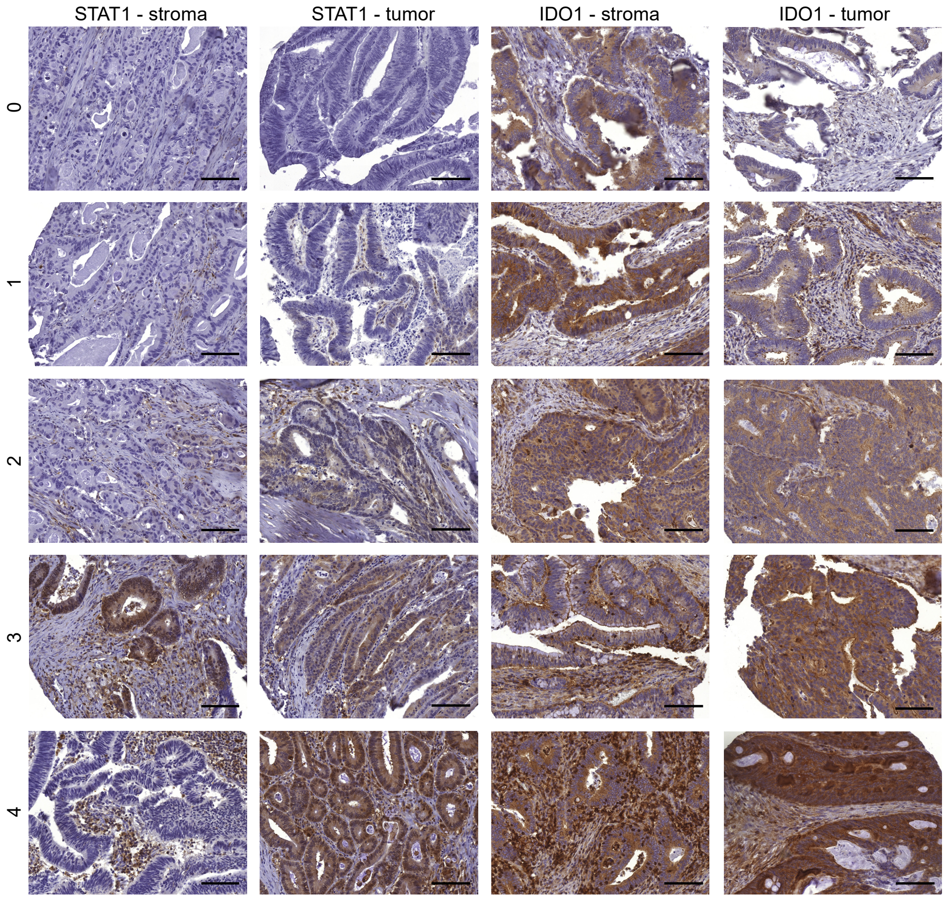

Supplementary figure 6: Representative IHC stainings for STAT1 and IDO1 on human CRC biopsies. Corresponding expression scores (1-4) are indicated. Scale bars indicate 100µm.

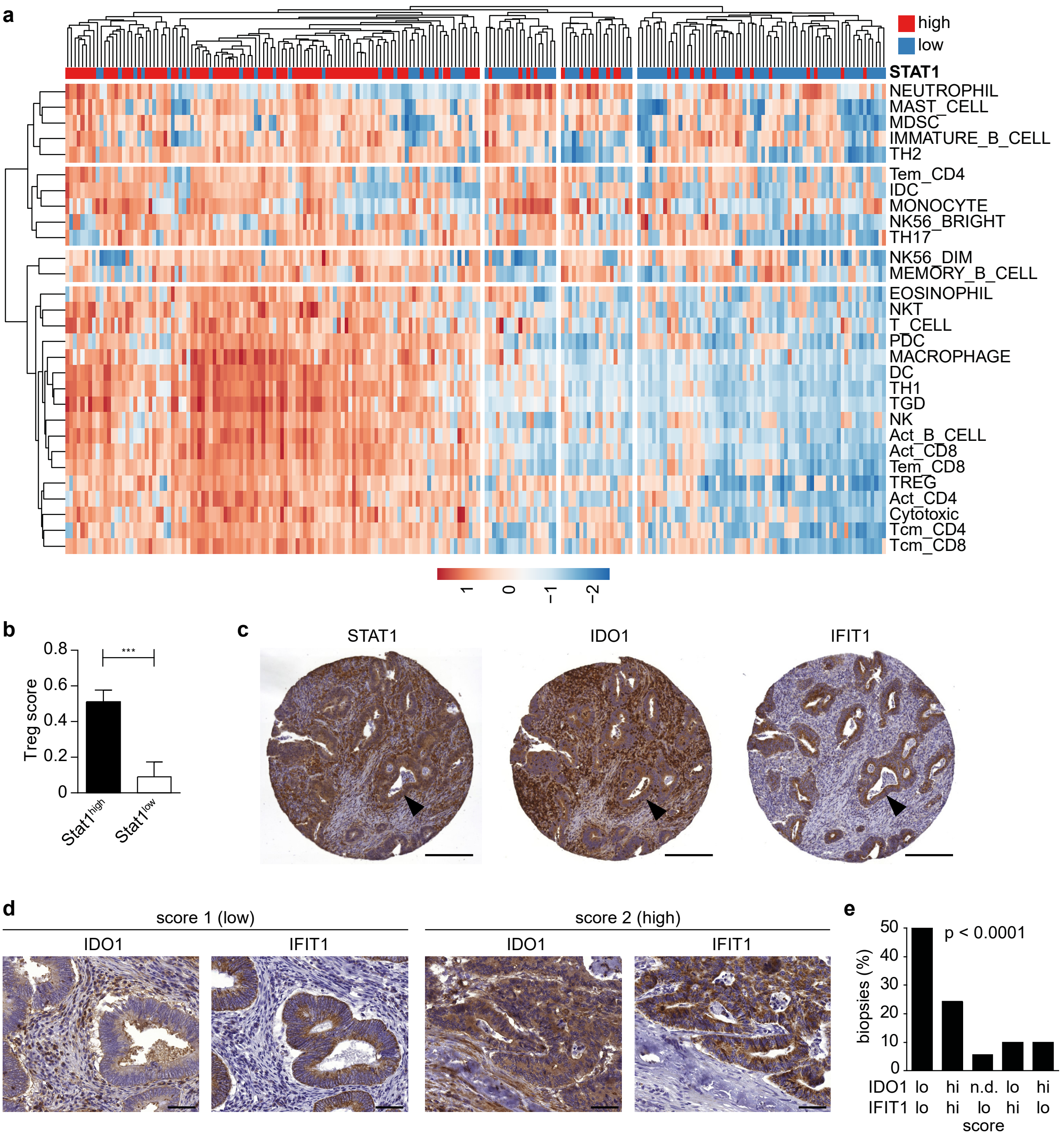

Supplementary figure 7: Ifit1 is a surrogate marker for Stat1-Ido1 expression in neoplastic epithelial cells of CRC. (a) Immunophenotyping of Stat1$^{high/low}$ CRC using immune cell metagene expression signatures and TCGA data. The median expression was used for stratification. (b) Assessment of Treg infiltration in Stat1$^{high/low}$ CRC using immunophenotypes. (c) IHC staining for STAT1, IDO1 and IFIT1 on consecutive TMA sections of human CRC. A tubular structure with cancer cells showing co-expression of STAT1, IDO1 and IFIT1 is indicated by arrowheads (scale bars 200µm). (d) IHC for IDO1 and IFIT1 on human CRC samples used for scoring of protein expressions. Note that IFIT1 is almost absent in the CRC stroma. Scale bars indicate 50µm. (e) Scoring results of 70 human CRC biopsies demonstrating a strong correlation between IDO1 and IFIT1 expressions in cancer cells. hi: high expression; lo: low expression; n.d.: not detectable. Bars represent mean +/- SEM.

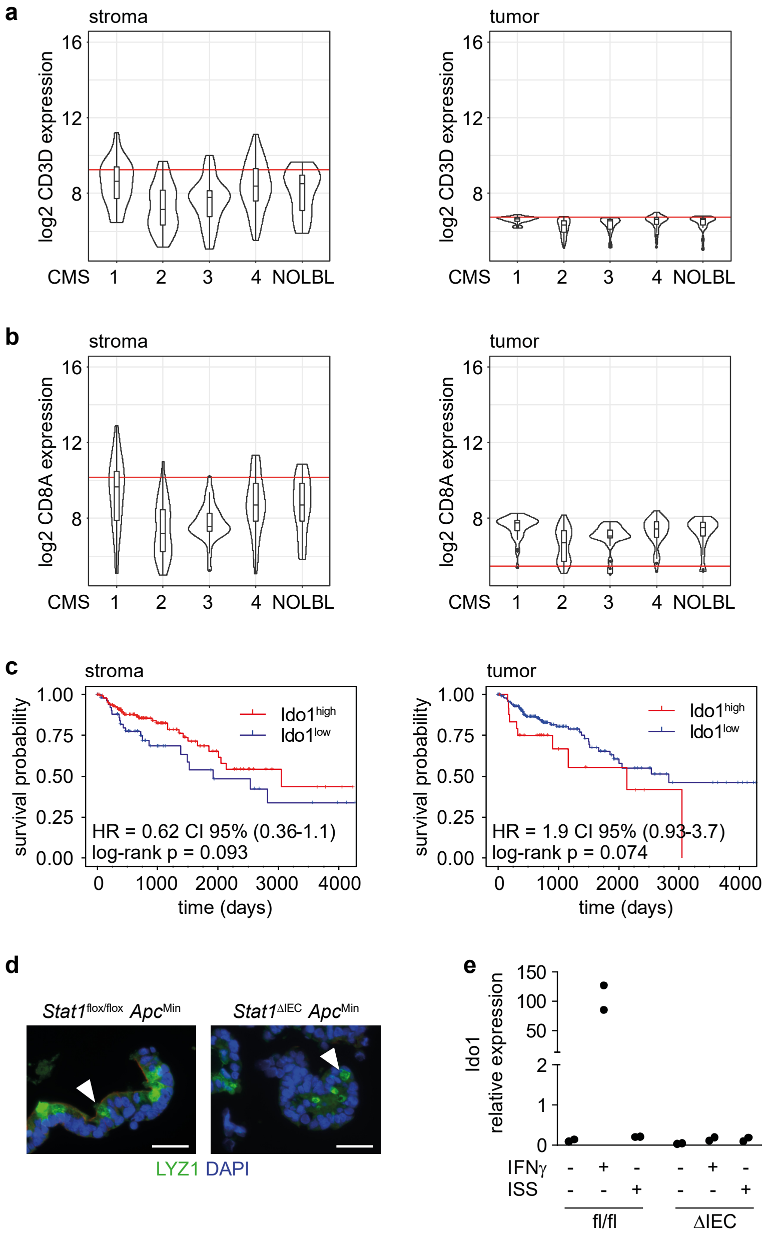

Supplementary figure 8: Validation of CRC TCGA expression data after transcriptome deconvolution with the DeMixT algorithm and patient survival curves for Ido1 expression in stroma cells or neoplastic epithelial cells. (a, b) Violin plots for expression of CD3 and CD8 T cell markers after stratification of CRC into consensus molecular subtypes 1-4. (c) Patient survival curves for Ido1 after transcriptome deconvolution. (d) IF showing LYZ1^{+} Paneth cells in organoids of *Stat1*$^{flox/flox}$ *Apc*^{Min} and *Stat1*$^{\Delta IEC}$ *Apc*^{Min} mice. Scale bars indicate 20µm. (e) qPCR for Ido1 mRNA expression after stimulation with IFNγ or the TLR9 ligand ISS-DNA in intestinal organoids of *Stat*1$^{flox/flox}$ and *Stat1*$^{\Delta IEC}$ mice (2 technical replicates).

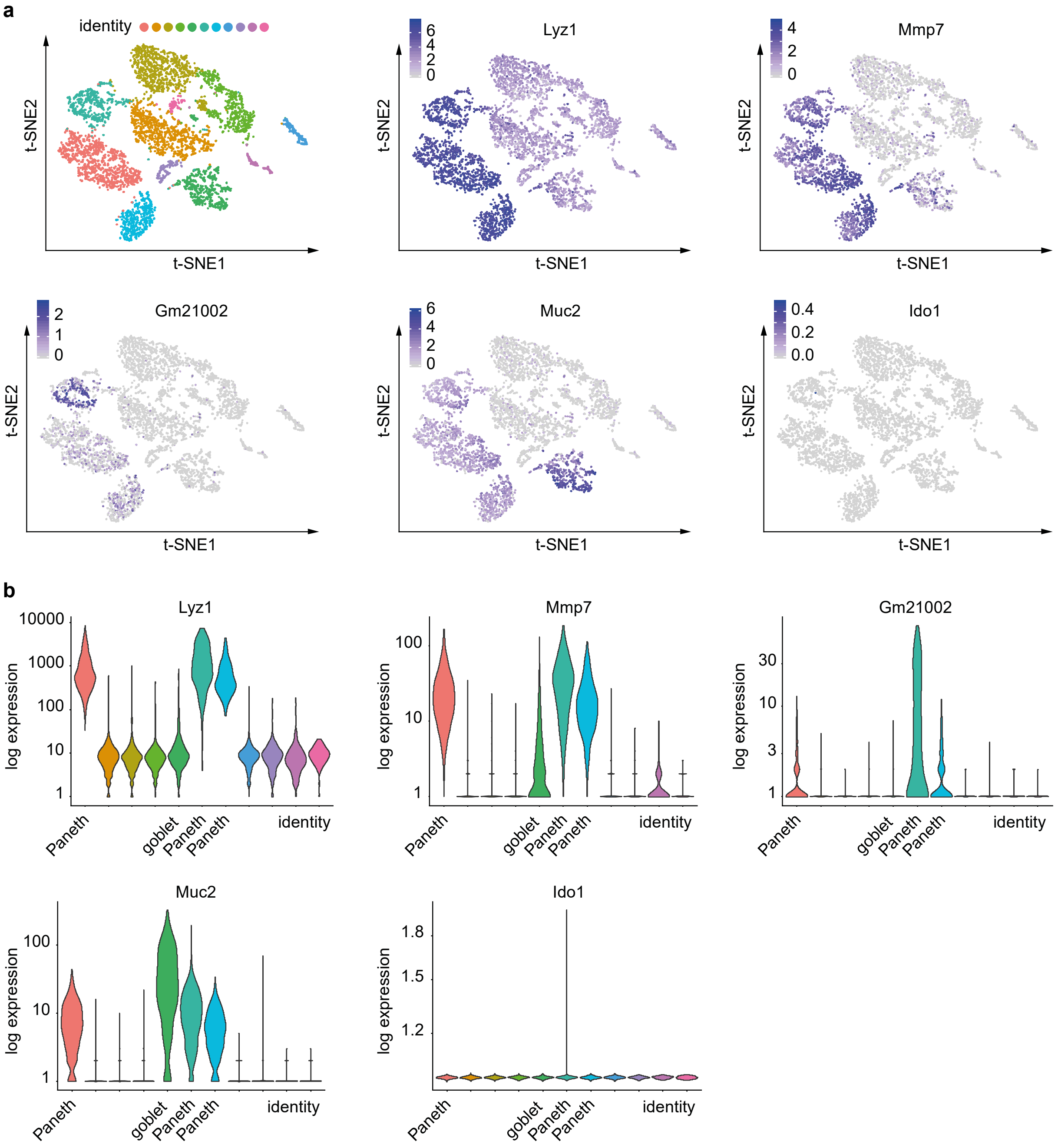

Supplementary figure 9: Expression of Ido1 mRNA in small intestinal epithelial cells. (a) t-SNE analysis was used to visualize cell type clusters (color coding) of 10.396 single cells, sequenced from small intestinal epithelium of C57BL/6 mice. Marker genes for Paneth and goblet cells were used to identify epithelial cell subsets with Ido1 expression. (b) Corresponding violin plots for expression of cell identity markers and Ido1. Single-cell RNA-seq data from Haber et al., Nature, 2017 (GEO database: GSE92332).

Supplementary tables

Supplementary table 1: Correlation analysis between Stat1 and bulk gene expression in TCGA data of human CRC identified 529 co-expressed genes with a Spearman score > 0.5.

Score	Genes
> 0.8	STAT1, GBP4, PARP9, GBP1, IFIT3, PARP14, UBE2L6, CXCL10
> 0.7	CXCL9, IDO1, GBP5, LAG3, TYMP, CD274, IRF1, CIITA, XAF1, LOC400759, SLC15A3, TAP1, APOBEC3G, SAMD9L, CD74, NKG7, IFNG, IFIT2, OAS2, IL2RB, HLA-DRA, CCL5, PDCD1, HLA-DPA1, TNFSF13B, TBX21, HLA-DMB, APOL3, WARS, TIGIT, IL18BP, IL12RB1, CD8A, CCR5, SLA2, AIM2, HLA-DPB1, ICAM1, JAKMIP1, IFI44L, IL21R, CMPK2, C1QA, ZBED2, RARRES3, C1QB
> 0.6	KLHDC7B, CRTAM, DOK2, CD2, PRF1, IRF9, GZMH, PSMB9, SH2D1A, LAP3, DDX60, HLA-DOA, CCL4, ITGAL, DDX58, TAP2, SIGLEC1, HLA-DMA, SP140, TNFRSF9, FAM26F, SIRPG, CTLA4, STX11, IFIT5, MYO1G, HAVCR2, HCST, FASLG, C1QC, BST2, PDCD1LG2, APOL2, RSAD2, CD96, SP110, ZNF683, PYHIN1, IL2RA, CXCL13, SLAMF8, STAT2, CD3E, CXCR6, IFI30, TRIM22, FCGR3A, ICOS, BTN3A1, ARHGAP9, CD7, CXCL11, BTN3A3, GZMA, FYB, LILRB2, HLA-E, CD86, CTSW, CD3G, CYTH4, LCP2, SLFN12L, KLRD1, WAS, ARHGAP30, GNGT2, APOBEC3H, PTPRC, CD80, LILRB4, CD247, DTX3L, SAMHD1, CMKLR1, OAS3, HLA-DRB1, TNFRSF18, CXCR2P1, CD226, SLA, ABI3, NLRC5, IFI44, RASAL3, FMNL1, CD3D, FPR3, C19orf38, GNLY, HAPLN3, GIMAP4, GPR174, TLR8, SPI1, MX1, CLEC7A, FCGR1B, CD300LF, IFIH1, SASH3, AIF1, DOCK2, ITGB2, ITK, LILRB3, FAM78A, CSF1, GPR171, FCER1G, NCF1, CD53, CCL8, KLRK1, GBP2, C5orf56, SAMSN1, SIGLEC10, UBASH3A, MYO1F, GZMK, LCP1, THEMIS, SP100, MAP4K1, RNF213, ARHGAP25, GIMAP5, MMP25, APBB1IP, HK3, CYBB, LAIR1, SLAMF7, LILRB1, APOL6, ADAR, IL10RA, CD163, TBC1D10C, CD300A, IFIT1, TRAF3IP3, GJD3, LTA, PLEK, MS4A6A, EOMES, PIK3CD, NLRC3, FCGR1A, CD84, IKZF1, GBP6, C1orf162, NCKAP1L, FCGR1C, CD4, HLA-DRB6, STAT4, HLA-DQA1, IL4I1, LRRC25, RASGRP1, TYROBP, UBD, FCRL6, ACAP1, HCLS1, LAPTM5, TFEC, C17orf87, ZNF831, CCR1, TRAFD1, MNDA, FOXP3, CD48, TRPV2, SAMD9, C3AR1, PARVG, MS4A4A, DOCK10, PTPRCAP, HCK, APOL1, GIMAP1, PPP1R16B, PIK3R5, TNFAIP8L2, IGSF6, FAM20A, CASS4, LSP1, SIGLEC7, NFAM1, CLIC2, CD14, PLA2G7, P2RY10, EPSTI1, SRGN, CST7, RASSF5, PLEKHO2, SLAMF1, RHOH, LST1, HLA-DRB5
> 0.5	GVIN1, PLA2G2D, ABCD2, NCF2, GZMM, EVI2A, GIMAP7, P2RX7, SPN, FAM49A, FAM115C, MPEG1, CLEC2B, FGR, DPYD, TRAT1, SIRPB2, UBASH3B, RASGRP3, TAGAP, CD38, GIMAP6, USP18, CD6, PRKCB, CD244, MAFB, DTHD1, LILRA6, CD37, ETV7, SIGLEC9, IL18RAP, CCR8, P2RY13, ISG15, BTK, CLEC12A, SFMBT2, IL15RA, RCSD1, GPR141, CD52, APOL4, VAMP5, ADAP2, CEACAM4, PTAFR, CSF1R, ITGAX, ST8SIA4, TMEM140, TARP, IL18R1, EVI2B, CHST11, GMFG, NCR1, KIAA0748, MEFV, FGD2, JAK2, NCF1B, MS4A7, CSF2RB, SELPLG, PSTPIP1, VSIG4, CD27, CD209, SLC1A3, APOE, BTN3A2, LILRB5, HLA-DPB2, SH2B3, GPR65, FGL2, TIFAB, TNFSF14, SLC2A5, CLEC4E, KLHL6, NLRC4, CLEC4A, CSF3R, FCGR2A, CD28, NCF1C, KIR2DL4, JAK3, GGTA1, CYSLTR2, TMEM149, SIGLEC5, ARHGAP15, PLEKHO1, IL9R, ODF3B, FCGR2C, GPR84, HLA-DOB, PML, PARP12, TLR1, TNFAIP2, BATF2, CD200R1, BATF3, SERPING1, TNFRSF8, CTSL1, GIMAP8, HPS5, MRC1, WDFY4, LY96, ARHGEF6, CCL18, RAB42, PILRA, CXorf21, EVL, March1, CD72, HLA-C, LILRA5, SIGLEC8, LY9, SELL, EIF2AK2, ZBP1, FLI1, KYNU, PIP4K2A, SUCNR1, IFI16, CCR2, LOC100233209, ADORA2A, GPNMB, HTRA4, ITGAM, TLR7, LIMD2, SPOCK2, EMR2, ZBTB32, DNAJC5B, PLXNC1, GAB3, KCNK13, HRH2, CD69, MYO5A, IFI35, FCGR2B, LOC154761, MX2, ADAMTS2, KIAA1949, MIAT, DOK3, IFI6, DRAM1, FAM179A, CECR1, TRIM69, FPR1, CFH, KMO, C10orf128, CYLD, SECTM1, CFLAR, HVCN1, C17orf60, CLEC6A, PIK3CG, HLA-DQB2, ACP5, GPR68, CD33, BCL2A1, ZAP70, IKZF3, HERC6, CD300C, HLA-DQA2, PRAM1, PIK3AP1, XCL2, IL10, ZNF804A, C5orf20, CCL3, TLR6, WIPF1, APOBEC3D, SDS, HIVEP3, CLEC10A, CCDC88A, BIRC3, LRRC8C, P2RY6, LAT2, C1orf38, LAMP3, GNB4, ADORA3, HLA-F, PTCRA, SLC31A2, NFATC1, LAX1, SERPINB9, TSPAN4, LILRA1, DPEP2, STAB1, EBI3, TNFRSF4, CLIP4, TTC24, PIK3R6, TLR2, SYTL3, CCL13, APOC1, NRP1, OPRL1, CLEC4D, RTP4, CD40, GM2A, PDE6G, EMR1, MSR1, TGM2, ZNF80, HSD11B1, CCL4L2, FLVCR2, SOCS1, SLFN5, VNN2, LOC400696, ARL4C, PTPN22, XRN1, KLRC1, HSD17B14, ALOX5AP, B2M, OSCAR, FCGR3B, PSAP, FLT3LG, GBP7, PLEKHF1, TGFB1, CEP170, XIRP1, SMTNL1

Supplementary table 2: Clinico-pathological features of 149 human CRC patients. p: p value.

	STAT1 Stroma					p
	**0 (%)**	**1 (%)**	**2 (%)**	**3 (%)**	**4 (%)**	
**Number of patients**	27	44	31	36	11	
**Median age (y)**	64,1	66,7	66,6	64,5	64,2	0,732
**Min. - max. age (y)**	41,1-78	41,3-77,9	43,8-76,8	30,0-77,4	46-70,7	
**Female/male**	48,1/51,9	50,0/50,0	51,6/48,4	44,4/55,6	45,5/54,5	0,979
**T category**						0,580
**-T3**	77,8	84,1	83,9	83,3	100,0	
**-T4**	22,2	15,9	16,1	16,7	0,0	
**Grading**						0,224
**- G1/G2**	70,4	86,4	83,9	80,6	100,0	
**- G3/G4**	29,6	13,6	16,1	19,4	0,0	
**Localisation**						0,969
**- Caecum**	11,1	13,6	9,7	13,9	9,1	
**- C. ascendens**	18,5	6,8	12,9	8,3	18,2	
**- Right flexure**	11,1	6,8	3,2	5,6	9,1	
**- C. transversum**	11,1	4,5	16,1	8,3	18,2	
**- Left flexure**	3,7	11,4	6,5	8,3	9,1	
**- C. descendens**	11,1	13,6	16,1	8,3	0,0	
**- Sigmoid**	33,3	43,2	35,5	47,2	36,4	

	STAT1 Tumor					p
	**0 (%)**	**1 (%)**	**2 (%)**	**3 (%)**	**4 (%)**	
**Number of patients**	52	29	21	19	28	
**Median age (y)**	64,6	64,5	67,3	66,7	61,3	0,186
**Min. - max. age (y)**	35,4-78,0	46-77,7	42,5-76,2	59,1-76,8	30-77,4	
**Female/male**	55,8/44,2	27,6/72,4	38,1/61,9	57,9/42,1	57,1/42,9	0,072
**T category**						0,066
**-T3**	73,1	93,1	95,2	89,5	82,1	
**-T4**	26,9	6,9	4,8	10,5	17,9	
**Grading**						0,96
**- G1/G2**	80,8	82,8	85,7	78,9	85,7	
**- G3/G4**	19,2	17,2	14,3	21,1	14,3	
**Localisation**						0,211
**- Caecum**	13,5	10,3	14,3	0	17,9	
**- C. ascendens**	13,5	6,9	0	21,1	14,3	
**- Right flexure**	9,6	0	0	5,3	14,3	
**- C. transversum**	7,7	6,9	9,5	10,5	17,9	
**- Left flexure**	11,5	10,3	4,8	5,3	3,6	
**- C. descendens**	13,5	13,8	14,3	0	10,7	
**- Sigmoid**	30,8	51,7	57,1	57,9	21,4	

	IDO1 Stroma					p
	**0 (%)**	**1 (%)**	**2 (%)**	**3 (%)**	**4 (%)**	
**Number of patients**	12	30	44	41	22	
**Median age (y)**	64,1	67,1	64	64,2	63,1	0,232
**Min. - max. age (y)**	41,1-74,4	46,3-78	42,5-77,2	30-77,4	45,9-77,9	
**Female/male**	58,3/41,7	40/60	56,8/43,2	39/61	54,5/45,5	0,361
**T category**						0,909
**-T3**	75	86,7	84,1	82,9	86,4	
**-T4**	25	13,3	15,9	17,1	13,6	
**Grading**						0,278
**- G1/G2**	100	90	77,3	80,5	77,3	
**- G3/G4**	0	10	22,7	19,5	22,7	
**Localisation**						0,262
**- Caecum**	8,3	3,3	18,2	12,2	13,6	
**- C. ascendens**	16,7	20	2,3	7,3	22,7	
**- Right flexure**	0	3,3	4,5	14,6	4,5	
**- C. transversum**	16,7	13,3	4,5	14,6	4,5	
**- Left flexure**	8,3	13,3	6,8	4,9	9,1	
**- C. descendens**	8,3	10	20,5	7,3	4,5	
**- Sigmoid**	41,7	36,7	43,2	39	40,9	

	IDO1 Tumor					p
	**0 (%)**	**1 (%)**	**2 (%)**	**3 (%)**	**4 (%)**	
**Number of patients**	10	34	48	47	10	
**Median age (y)**	67,1	61,1	65,84	66,1	65,4	0,253
**Min. - max. age (y)**	52,5-78	30-77,2	35,4-77,7	43,2-77,4	41,5-75,4	
**Female/male**	50/50	52,9/47,1	47,9/52,1	48,9/51,1	30/70	0,799
**T category**						0,329
**-T3**	80	79,4	89,6	78,7	100	
**-T4**	20	20,6	10,4	21,3	0	
**Grading**						0,438
**- G1/G2**	70	91,2	81,3	78,7	90	
**- G3/G4**	30	8,8	18,8	21,3	10	
**Localisation**						0,693
**- Caecum**	10	8,8	12,5	17	0	
**- C. ascendens**	20	14,7	8,3	10,6	10	
**- Right flexure**	0	8,8	4,2	10,6	0	
**- C. transversum**	30	14,7	8,3	6,4	0	
**- Left flexure**	10	2,9	8,3	10,6	10	
**- C. descendens**	10	14,7	10,4	10,6	10	
**- Sigmoid**	20	35,3	47,9	34	70	
