# Supplementary material for: IDO1+ Paneth cells promote immune escape of colorectal cancer
Source: Commun Biol. 2020 May 22;3:252. doi: 10.1038/s42003-020-0989-y (PMC7244549; doi:10.1038/s42003-020-0989-y)
Supplement: Supplementary file 6 — Description of Additional Supplementary Files [file 42003_2020_989_MOESM6_ESM.pdf]

Description of Additional Supplementary Files

File Name: **Supplementary Data 1**

Description: Genes, downregulated > 2-fold in Stat1$^{\Delta IEC}$ Apc^{Min} tumors (compared to Stat1$^{flox/flox}$ Apc^{Min} tumors). logFC: log2 fold change; FDR: false discovery rate (< 0.005).

File Name: **Supplementary Data 2**

Description: Genes, upregulated > 2-fold in Stat1$^{\Delta IEC}$ Apc^{Min} tumors (compared to Stat1$^{flox/flox}$ Apc^{Min} tumors). logFC: log2 fold change; FDR: false discovery rate (< 0.005).

File Name: **Supplementary Data 3**

Description: GO term enrichment analysis of RNA-seq data from Stat1$^{flox/flox}$ Apc^{Min} and Stat1$^{\Delta IEC}$ Apc^{Min} colon tumors (FDR < 0.005). p: p value, q: FDR q-value.

File Name: **Supplementary Data 4**

Description: GO term enrichment analysis of 529 genes, co-expressed with Stat1 in human CRC with a Spearman score > 0.5. p: p value.
