# Supplementary material for: IDO1+ Paneth cells promote immune escape of colorectal cancer
Source: Commun Biol. 2020 May 22;3:252. doi: 10.1038/s42003-020-0989-y (PMC7244549; doi:10.1038/s42003-020-0989-y)
Supplement: Supplementary file 7 — Reporting Summary [file 42003_2020_989_MOESM7_ESM.pdf]

nature research

Corresponding author(s): Robert Eferl
Last updated by author(s): Apr 10, 2020

Reporting Summary

Nature Research wishes to improve the reproducibility of the work that we publish. This form provides structure for consistency and transparency in reporting. For further information on Nature Research policies, see Authors & Referees and the Editorial Policy Checklist.

Statistics

For all statistical analyses, confirm that the following items are present in the figure legend, table legend, main text, or Methods section.

n/a	Confirmed	
☐	☒	The exact sample size (*n*) for each experimental group/condition, given as a discrete number and unit of measurement
☐	☒	A statement on whether measurements were taken from distinct samples or whether the same sample was measured repeatedly
☐	☒	The statistical test(s) used AND whether they are one- or two-sided<br>*Only common tests should be described solely by name; describe more complex techniques in the Methods section.*
☒	☐	A description of all covariates tested
☐	☒	A description of any assumptions or corrections, such as tests of normality and adjustment for multiple comparisons
☐	☒	A full description of the statistical parameters including central tendency (e.g. means) or other basic estimates (e.g. regression coefficient) AND variation (e.g. standard deviation) or associated estimates of uncertainty (e.g. confidence intervals)
☒	☐	For null hypothesis testing, the test statistic (e.g. *F*, *t*, *r*) with confidence intervals, effect sizes, degrees of freedom and *P* value noted<br>*Give P values as exact values whenever suitable.*
☒	☐	For Bayesian analysis, information on the choice of priors and Markov chain Monte Carlo settings
☒	☐	For hierarchical and complex designs, identification of the appropriate level for tests and full reporting of outcomes
☒	☐	Estimates of effect sizes (e.g. Cohen's *d*, Pearson's *r*), indicating how they were calculated

Our web collection on statistics for biologists contains articles on many of the points above.

Software and code

Policy information about availability of computer code

Data collection	No software was used to collect data in this study.
Data analysis	TopHat2 algorithm was used to align raw RNA-seq data, differentially expressed genes were identified using DeSeq2 algorithm, Gene Ontology enrichment analyses were performed using GOrilla software, DeMixT algorithm was used to separate tumor cell-intrinsic and stromal expression of Ido1 in TCGA data, correlation analyses of TCGA data were calculated using cor function of R3.2.1 software and visualization was performed using corrplot and ggplot2 packages.<br>Pre-processed droplet-based scRNA-seq data sets from Haber et. al., Nature (2017) (GEO; GSE92332) were re-analyzed using the R package "Seurat".

For manuscripts utilizing custom algorithms or software that are central to the research but not yet described in published literature, software must be made available to editors/reviewers. We strongly encourage code deposition in a community repository (e.g. GitHub). See the Nature Research guidelines for submitting code & software for further information.

Data

Policy information about availability of data

All manuscripts must include a data availability statement. This statement should provide the following information, where applicable:

- Accession codes, unique identifiers, or web links for publicly available datasets
- A list of figures that have associated raw data
- A description of any restrictions on data availability

RNA sequencing data were deposited in ArrayExpress database, accession number E-MTAB-5083. Previously generated single-cell RNA sequencing data analysed here can be found in GEO (GSE92332). Processed (MapSplice aligned, RSEM quantified and upper-quartile normalization standardized; Level 3 RnaSeqV2) RNA sequencing data of the COADREAD dataset were obtained from The Cancer Genome Atlas (TCGA) database. All other data that support the findings of this study are available from the corresponding author upon reasonable request.

Field-specific reporting

Please select the one below that is the best fit for your research. If you are not sure, read the appropriate sections before making your selection.

☒ Life sciences ☐ Behavioural & social sciences ☐ Ecological, evolutionary & environmental sciences

For a reference copy of the document with all sections, see nature.com/documents/nr-reporting-summary-flat.pdf

Life sciences study design

All studies must disclose on these points even when the disclosure is negative.

Sample size	No sample size estimation was performed.
Data exclusions	Samples were excluded as outliers according to Grubbs' test (α = 0.05).
Replication	Experimental findings were reproducible in repetitions of measurements/analyses.
Randomization	Experiments were performed and analyzed in a blinded, randomized manner.
Blinding	Experiments were performed and analyzed in a blinded, randomized manner.

Reporting for specific materials, systems and methods

We require information from authors about some types of materials, experimental systems and methods used in many studies. Here, indicate whether each material, system or method listed is relevant to your study. If you are not sure if a list item applies to your research, read the appropriate section before selecting a response.

Materials & experimental systems

n/a	Involved in the study	
☐	☒	Antibodies
☐	☒	Eukaryotic cell lines
☒	☐	Palaeontology
☐	☒	Animals and other organisms
☒	☐	Human research participants
☐	☒	Clinical data

Methods

n/a	Involved in the study	
☒	☐	ChIP-seq
☐	☒	Flow cytometry
☒	☐	MRI-based neuroimaging

Antibodies

Antibodies used	CD8a (Biolegend, 100728), CD45 (Biolegend, 103128), CD4 (Biolegend, 100408), CD3e (eBioscience, 35-0031-82), CD25 (eBioscience, 25-0251-81), FOXP3 (Biolegend, 320011), Granzyme B (Biolegend, 515405 and Abcam, ab4059), BrdU (BrdU In-Situ Detection Kit, Becton Dickinson, 550803), Cleaved Caspase 3 (Cell signaling, 9661), Endomucin (eBioscience, 14-5851-82), Gr1 (Serotec, MCA771GA), Ido1 (Biolegend, 122402 and 654002), iNOS (Biolegend, 610431), Ki67 (Novocastra, NCL-KI67-P), Lysozyme (Dako, A009902), p-Stat1 (Cell Signaling, 9167S), p-Stat3 (Cell signaling, 9145), Stat1 (Santa Cruz, sc-592 and Cell Signaling, 14994), Stat3 (Santa Cruz, sc-7179), Synaptophysin (GeneTex, GTX100865), Ifit1 (Sigma Aldrich, HPA055380), GFP (Roche, 11814460001), RFP (Rockland antibodies & assays, 600-401-379S), CD3 (Neomarker RM9107), MMP7 (Cell Signaling, 3801), E-cadherin (Abcam, 11512).
Validation	CD8a, CD45, CD4, CD3e, CD25 and FOXP3 antibodies are standard antibodies for FACS analysis. We provide FACS profiles showing the gating strategy in Supplementary fig. 3c. Antibodies for Granzyme B, BrdU, Cleaved Caspase 3, Gr1, iNOS, Ki67 are standard antibodies for IHC/IF. Specificity was evaluated by the staining pattern in most cases (e.g. Ki67 provided positive signals in cells of the intestinal crypts as expected, Supplementary fig. 1g). The endomucin antibody stained specifically blood vessels as expected. Specificity of Ido1 staining in Paneth cells was validated by in situ hybridization for Ido1 RNA. The same cells were positive for Ido1 protein and RNA (Figure 2a). Antibodies for Stat1 and p-Stat1 were validated by IHC staining of Stat1 knock-out tissue (e.g. Supplementary fig. 1c). Antibodies for Stat3 and p-Stat3 have been validated by us in several manuscripts using Stat3 knock-out tissue (e.g. Musteanu et al. Gastroenterolgy 2010, https://doi.org/10.1053/j.gastro.2009.11.049). Antibodies for Lysozyme and Synaptophysin were validated by IHC staining of intestinal tissue. Lysozyme stained specifically granule-containing Paneth cells in the intestinal crypts. Synaptophysin antibody displayed specific staining of cells with enteroendocrine morphology. The Ifit1 antibody stained specifically the neoplastic epithelium of CRC samples but not the tumor stroma (Supplementary fig. 7c) as expected. GFP and RFP antibodies stained specifically cancer cells harboring corresponding transgenes but not non-transfected cells. The MMP7 antibody stained the same granule-containing cells as Lysozyme as expected for a Paneth marker. The E-cadherin antibody provided a specific membrane staining as expected.

Eukaryotic cell lines

Policy information about cell lines

Cell line source(s)	MC38, C57BL/6 derived.
Authentication	none of the cell lines were authenticated.
Mycoplasma contamination	cells were not tested for mycoplasma contamination.
Commonly misidentified lines (See ICLAC register)	no misidentified cell lines were used.

Animals and other organisms

Policy information about studies involving animals; ARRIVE guidelines recommended for reporting animal research

Laboratory animals	Mice were kept on a C57BL/6 genetic background. For analysis of tumor development, 4 month old male and female mice were used for experiments. Intestinal epithelial cells were isolated from 10-12 week old male and female mice. For subcutaneous injection of tumor cells 8-9 week old male C57BL6/J and NSG mice were used.
Wild animals	No wild animals were used.
Field-collected samples	No field-collected samples were used.
Ethics oversight	All mouse experiments were performed in accordance with Austrian and European laws and with the general regulations specified by the Good Science Practices guidelines of the Medical Universities of Vienna and Innsbruck.

Note that full information on the approval of the study protocol must also be provided in the manuscript.

Clinical data

Policy information about clinical studies

All manuscripts should comply with the ICMJE guidelines for publication of clinical research and a completed CONSORT checklist must be included with all submissions.

Clinical trial registration	Patient material from the Austrian Breast and Colorectal Cancer Study 91 (ABCSG trial 91, NCT00309543) was used. All patients provided written consent and the study was approved by the ethics committees at the participating institutions.
Study protocol	The study protocol is described in Schippinger et al. 2007. A prospective randomised phase III trial of adjuvant chemotherapy with 5-fluorouracil and leucovorin in patients with stage II colon cancer. British Journal of Cancer 97, 1021 – 1027. Doi: 10.1038/sj.bjc.6604011.
Data collection	Data collection is described in Schippinger et al. 2007. A prospective randomised phase III trial of adjuvant chemotherapy with 5-fluorouracil and leucovorin in patients with stage II colon cancer. British Journal of Cancer 97, 1021 – 1027. Doi: 10.1038/sj.bjc.6604011. To be eligible for this study, patients had to fulfil the following inclusion criteria: written informed consent to participate in this trial, histologically proven stage II colon carcinoma according to the UICC (T3-T4, N0, M0), potentially curative resection without gross or microscopic evidence of residual disease, age between 18 and 80 years, World Health Organization (WHO) performance status of 0 or 1, absence of severe concomitant disease and other malignancies, and adequate bone marrow, renal and hepatic function. The following were applied as exclusion criteria: prior or concomitant chemotherapy, immunotherapy or radiotherapy, and carcinoma of the rectum defined as tumour below the anatomical rectosigmoidal borderline or within 16 cm from the anal verge measured by a non-flexible rectoscope. Other exclusion criteria included the presence of metastasis or an interval exceeding 42 days between surgery and the start of adjuvant therapy. Written informed consent was obtained from all patients participating in this trial. Monitoring visits were performed regularly at the participating centres to inspect the original data concerning eligibility and to review documented chemotherapy and follow-up data.
Outcomes	After receiving permission from the Austrian Breast and Colorectal Cancer Study Group (https://www.abcsg.org), we IHC-stained the tissue samples of the ABCSG trial 91 for STAT1 and IDO1. We pre-defined the primary outcome measures in the following way: assessment of a correlation between STAT1-IDO1 expression in the neoplastic epithelium with prognosis of colon cancer patients. Assessment of all other possible correlations were pre-defined as secondary outcome measures. Measures were assessed by scoring of staining intensities in the neoplastic epithelium and the tumor stroma (scores 1-4; 1: no staining, 2: weak staining, 3: moderate staining, 4: strong staining) and chi-square testing for correlations with patient parameters.

Flow Cytometry

Plots

Confirm that:

☒ The axis labels state the marker and fluorochrome used (e.g. CD4-FITC).

☒ The axis scales are clearly visible. Include numbers along axes only for bottom left plot of group (a 'group' is an analysis of identical markers).

☒ All plots are contour plots with outliers or pseudocolor plots.

☒ A numerical value for number of cells or percentage (with statistics) is provided.

Methodology

Sample preparation	Intestinal tumors from single mice were pooled, minced and digested in 2 ml phosphate buffered saline containing 0.25% (v/v) fetal calf serum and 0,25% (w/v) collagenase IV (Life technologies, 17104-019) for 45’ at 37°C under shaking. After straining through a 70 µm mesh and washing twice with 30 ml phosphate buffered saline, cells were incubated with TruStain fcX (Biolegend, 101320) and Zombie Aqua Fixable Viability Kit (Biolegend, 423102).
Instrument	Data were collected using a FACS Fortessa (BD).
Software	Data were analyzed with FlowJo software.
Cell population abundance	No post-sort fractions were used.
Gating strategy	Gating strategy is exemplified in the Supplementary Information.

☒ Tick this box to confirm that a figure exemplifying the gating strategy is provided in the Supplementary Information.
